# Source matters: a survey of cost variation for fecal immunochemical tests in primary care

**DOI:** 10.1186/s12913-022-07576-4

**Published:** 2022-02-15

**Authors:** Jennifer Coury, Katrina Ramsey, Rose Gunn, Jon Judkins, Melinda Davis

**Affiliations:** 1 Oregon Rural Practice-based Research Network, Oregon Health & Science University, 3181 S.W. Sam Jackson Park Rd., Mail Code L222, Portland, Oregon 97239 USA; 2 OCHIN, Inc, Portland, USA; 3 Internal Medicine, Oregon Health & Science University, Portland, OR USA; 4 Department of Family Medicine, Oregon Health & Science University, Portland, OR USA; 5 OHSU-PSU School of Public Health, Oregon Health & Science University, Portland, OR USA

**Keywords:** Colorectal cancer screening, cancer screening outreach, Fecal immunochemical testing (FIT), Screening costs

## Abstract

**Background:**

Colorectal cancer (CRC) screening can improve health outcomes, but screening rates remain low across the US. Mailed fecal immunochemical tests (FIT) are an effective way to increase CRC screening rates, but is still underutilized. In particular, cost of FIT has not been explored in relation to practice characteristics, FIT selection, and screening outreach approaches.

**Methods:**

We administered a cross-sectional survey drawing from prior validated measures to 252 primary care practices to assess characteristics and context that could affect the implementation of direct mail fecal testing programs, including the cost, source of test, and types of FIT used. We analyzed the range of costs for the tests, and identified practice and test procurement factors. We examined the distributions of practice characteristics for FIT use and costs answers using the non-parametric Wilcoxon rank-sum test. We used Pearson’s chi-squared test of association and interpreted a low *p*-value (e.g. < 0.05) as evidence of association between a given practice characteristic and knowing the cost of FIT or fecal occult blood test (FOBT).

**Results:**

Among the 84 viable practice survey responses, more than 10 different types of FIT/FOBTs were in use; 76% of practices used one of the five most common FIT types. Only 40 practices (48%) provided information on FIT costs. Thirteen (32%) of these practices received the tests for free while 27 (68%) paid for their tests; median reported cost of a FIT was $3.04, with a range from $0.83 to $6.41 per test. Costs were not statistically significantly different by FIT type. However, practices who received FITs from manufacturer’s vendors were more likely to know the cost (*p* = 0.0002) and, if known, report a higher cost (*p* = 0.0002).

**Conclusions:**

Our findings indicate that most practices without lab or health system supplied FITs are spending more to procure tests. Cost of FIT may impact the willingness of practices to distribute FITs through population outreach strategies, such as mailed FIT. Differences in the ability to obtain FIT tests in a cost-effective manner could have consequences for implementation of outreach programs that address colorectal cancer screening disparities in primary care practices.

**Supplementary Information:**

The online version contains supplementary material available at 10.1186/s12913-022-07576-4.

## Background

Colorectal cancer (CRC) is the third leading cause of cancer death, and almost 53,000 people in the US are projected to die of colorectal cancer in 2021 [1]. Mortality and incidence rates continue to be high [2, 3] despite the availability of multiple effective screening modalities as recommended by the United States Preventive Services Task Force (USPSTF) [4, 5]. Rates of screening are still quite low in the US population and disparities persist (such as among rural residents and Medicaid enrollees), due in part to different adherence to screening guidelines [6, 7].

Expanding the use of fecal immunochemical testing (FIT) is a noninvasive and cost-effective approach to addressing disparities in CRC screening [8–12]. However, over 160 different types of FIT or fecal occult blood testing (FOBT) are approved for use by the FDA, which may lead to variation in FIT use by individual practices. Available FITs vary by clinical effectiveness (such as detection rates and positivity threshold), cost of FIT [13], and patient preferred features, such as using a single sample and probe/vial collection tubes [14]. Some literature indicates community-based primary care practices use a wide variety of FITs, many of which do not have strong evidence of efficacy [14, 15].

Distributing high quality FITs during clinic vists and through mailed FIT outreach are identified as important strategies to achieve CRC screening targets and to address CRC screening disparities [16–19]. Mailed FIT may also help overcome CRC screening delays caused by the COVID pandemic [20–22]. Surveys indicate patients might be more reluctant to be screened by endoscopy due to fears of COVID infection [21, 23] and mailing a test to people’s homes offers a non-visit-based way to reach people for screening [22, 24]. Despite evidence to support implementation of mailed FIT programs [18], they are not yet part of routine care.

Clinic readiness, provider receptivity to FIT, and other organizational factors (e.g., system affiliation, organizational partnerships, geography) are posited to influence the adoption of new interventions, including mailed FIT programs [25, 26]. The integrated-Promoting Action on Research Implementation in Health Services (i-PARIHS) framework posits that successful implementation is a function of facilitation interacting with recipients, intervention, and context [27]. While FIT cost is a characteristic of the intervention, clinic factors (e.g., the context of geographic location, practice size, CRC screening methods already used in the clinical practice) may dictate these costs. To implement a screening program such as mailed FIT, practices must consider many costs, such as FIT procurement, associated lab processing, and supportive outreach activities (e.g., in-clinic staffing for distribution, prompts, and reminder calls). In the US, while health insurance plans pay for the processing of the FITs, the costs for implementing outreach programs may be accrued by the clinical practice, the health system (such as a hospital system or a health maintenance organization), or the health insurance plan [28, 29].

While some literature examines the cost of implementing CRC screening programs, most of those studies focus on the labor and organizational costs that make up the vast majority of the expense of implementing such programs [30, 31]. Although studies have reported the overall cost of mailed FIT programs [32, 33], few have explored the cost of FIT procurement. Therefore, we undertook this survey to address the gap in knowledge about which factors actually influence primary care practices’ FIT choice and willingness to implement mailed FIT programs, with a primary goal of understanding the influence of cost. This manuscript reports on what contextual factors were associated with (a) FIT selection and (b) FIT costs. For example, do small, rural clinics pay more for FIT than large, urban clinics? Answers to these questions could be critical to informing interventions to increase the adoption of high quality FITs and mailed FIT implementation.

## Methods

This cross-sectional, survey-based study was approved by the Oregon Health & Science University Institutional Review Board (#17952). Participants reviewed an information sheet outlining the study purposes and risks and were instructed to contact study staff with questions; completion of the survey constituted informed consent.

### Participants and setting

To generate the list of eligible primary care practices, we created a listing of all practices involved in Oregon Rural Practice-based Research Network (ORPRN) research and technical assistance contracts over the past 3 years (*N* = 298). ORPRN is a practice-based research network that was established in 2002 to promote research, education, and community engagement activities in partnership with rural primary care practices [34]. After review by two members of the study team (MMD, RG), 13 of the 298 potential practices were excluded as they did not provide primary care services. Prior to data collection, in the fall/winter of 2018, a member of the study team called the remaining practices to verify information for the practice’s listed point of contact and to determine preferred method of survey delivery (email, mail, or fax). Thirty-three additional practices were excluded at this point, as they were healthcare systems with a single point of contact for multiple affiliated practices. This left a final count of 252 eligible primary care practices.

### Survey measures and data collection

We administered a multi-modal, cross-sectional survey to the 252 primary care practices to assess contextual factors that might impact the implementation of mailed FIT programs as informed by the i-PARIHS framework [25]. We determined a survey was the most practical way to get a broad response sample to capture information on the type of FIT, cost, source and practice characteristics to address our research questions. Survey questions were designed primarily using validated measures from existing instruments, including the Organizational Readiness to Implement Change (ORIC) [26] and the Change Process Capability Questionnaire (CPCQ) [35]. Survey items assessed practice readiness to implement the innovation (mailed FIT) and inner and outer context. Context questions included implementation climate and current quality improvement activities, practice size, ownership, federal designations, geographic location, and percent of Medicaid patients [36]. If a validated question was not available, questions were modeled after ones used in prior studies to assess clinic workflows [37–39].

The survey in its entirety was designed to take less than 15 min to complete. After a quality assurance check from internal study advisors, we pilot tested an initial version of the survey with three clinics and made minor changes based upon feedback (e.g., adding a small number of free text answer options) prior to deployment. The final 26-item “Understanding Practice Readiness to Increase Colorectal Cancer Screening via Direct Mail Programs” survey (see Additional file 1) was broken into 5 sections: practice-specific CRC screening data, FIT/FOBT use & costs, direct mail program use, general practice characteristics, and use of strategies to improve CRC screening rates. Most survey questions were fixed response items or used a likert scale; some items were answered using open ended text or numerical fields (e.g., number of clinicians). A full copy of the survey is available in supplementary materials.

The FIT/FOBT use and costs section included four questions addressing type of test used, where the tests were obtained, and cost of individual kits. In prior research about mailed FIT programs [29, 31], the research team discovered three primary sources of obtaining FITs: 1) supplied by the lab that would process the completed FITs (i.e., Lab); 2) obtained from the FIT manufacturer through a vendor (i.e., Vendor); or 3) supplied by a hospital system or other health network (i.e., Health system) of which the practice was affiliated. Our survey asked about both FIT and FOBT because some practices still use FOBT for CRC, but we have utilized “FIT” generically throughout the manuscript for clarity and simplicity.

The survey was launched in January 2018 and was administered by email (*N* = 173) and fax (*N* = 79), depending on stated preferences. If the point of contact (primarily administrative staff such as quality improvement leads or practice managers) had not returned the survey within 1 week, reminders were made by email or phone. Over the course of the next 7 months, up to 5 reminder contacts were made. In July 2018 the list of non-responding practices was reviewed with ORPRN’s regional practice facilitators [40, 41], who were asked to contact practices in their regions to encourage survey return. In the summer of 2019, a member of the research team re-contacted practices and obtained additional responses to the cost of FIT section.

### Data management and analysis

Practices were classified based on yes or no responses to the question, “Do you know how much a FIT/FOBT kit costs your practice?” We examined the distributions of practice characteristics using descriptive statistics, reported as mean and range, or as counts and percents for categorical variables. Practice size was based on the reported number of medical clinicians (MD, DO, NP, and/or PA); practice size categories were solo/partnership (1–2 clinicians), small to medium practice (3–10 clinicians), or large practice (> 10 clinicians). We determined geographic location as frontier, rural, or urban using the ZIP code from the practice’s physical address; classifications are provided by the Oregon Office of Rural Health [42]. Patient visits per week, percent of patients with Medicaid coverage, current CRC screening rate, number of ongoing quality improvement (QI) projects related to CRC screening, priorities and opinions, and source of FIT/FOBT kits were used as reported by respondents.

In response to the question regarding FIT/FOBT kit costs, if the answer was formulated as the cost per a certain number of kits (e.g. “$137 for 30 tests”), we calculated the cost of an individual test. Four practices reported costs that were outliers ($17–31 per FIT, compared with a maximum of $7 for the remainder). Analyses comparing costs by source were performed both with and without these four observations; results presented here omit those values as follow-up calls confirmed that one of these responses included lab processing in addition to the cost of the FIT itself, thus we suspect these four values were not comparable to other FIT costs.

When comparing proportions, we used Pearson’s chi-squared test of association and interpreted a low *p*-value (e.g. < 0.05) as evidence of association between a given practice characteristic and knowing the cost of FIT. We tested differences in the distributions of continuous variables (e.g. the cost per kit, given that a cost was reported) using the non-parametric Wilcoxon rank-sum test. We performed exploratory regression analyses (linear and logistic) to better understand some relationships, such as between practice size, source of FIT kits, knowing FIT cost, and (square-root transformed) cost. In the final analysis, practices obtaining FITs from either a lab or health system were combined into one category due to small sample size with similar cost patterns that would prevent any meaningful further sub-analysis. Statistical analyses were completed using Stata Statistical Software/IC Release 15 (StataCorp, LLC, 2017) and utilized the user-contributed *tabcount* command [43].

## Results

At the close of the survey in August 2018, 90 surveys had been returned from 84 practices (33% response rate). We compared characteristics of the 84 practices included in our analysis with non-responders. Based on ZIP codes, responding practices were more likely than non-respondents to be located in frontier (12% vs 3%, respectively) and rural (48% vs 41%) areas and less likely to be in urban areas (40% vs 57%; *p* = 0.004). More responders were Federally Qualified Health Centers, Rural Health Centers, or government-run (24% vs 15%) and fewer were affiliated with hospital or health systems (52% vs 62%) or were clinician-owned solo or group practices (17% vs 20%; *p* = .18 for overall distribution). Overall, the sample was similar enough to the overall list for our analyses.

Across the 84 survey respondents, more than 10 different types of FIT kits were in use, see Table 1. Five tests were used by more than 10% of the response sample, including: Hemosure® by Hemosure, Inc. (30%), OC Auto® by Polymedco (19%), Insure® by Clinical Genomics (13%), Hemoccult-ICT® by Beckman Coulter (12%) and OC-Light® iFOBT by Polymedco (11%). Three out of four practices (64 out of 84, 76%) used one of these more common FITs. In addition, some practices (*n* = 7) reported using multiple FIT tests simultaneously. Clarifying information from these practices suggested that FIT type depended on insurance coverage or whether the clinic was part of a health system partnered mailed screening outreach initiative.

**Table 1:** Type of FIT/FOBT and Reported Costs from 84 Practice Respondents

Kit Name	N^*^	%	Unknown cost, N	Free, N^*^	Purchase kits			
					Known Cost, N	Median ($)	Min.	Max.
Hemosure® One-Step iFOBT Test (Hemosure, Inc)	25	(30)	8	6	10^a^	3.43	0.83	6.41
OC Auto® FIT^**^ (Polymedco)	16	(19)	10	4	2	1.57	1.13	2.00
Insure® FIT (Clinical Genomics)	11	(13)	5	2	3	2.32	1.70	6.00
Hemoccult-ICT® (Beckman Coulter)	10	(12)	7	0	1^b^	0.99	0.99	0.99
OC-Light® iFOBT Test^**^ (Polymedco)	9	(11)	6	2	1	1.50		
McKesson Consult® FOBT	3	(4)	0	1	2	3.44	0.88	6.00
Seracult®	2	(2)	1	0	1	2.13		
QuickVue® iFOBT (Quidell)	2	(2)	2	0	0			
Lochness Medical®	1	(1)	0	0	1	5.00		
Rapid Response® FIT (BTNX Inc.)	1	(1)	0	0	1	3.75		
HemaPrompt®	1	(1)	0	0	1	1.41		
*Unknown*	11	(13)	11	0	0			
**Overall**	**84**	**(100)**	**44**	**13**	**27**	**3.04**	**0.83**	**6.41**

^a^One outlier ($18.40) omitted, and ^b^: Two outliers ($17.00 and $30.00 omitted); as detailed in “Methods” these responses likely include processing or are the cost per box rather than cost per unit

^*^Note that FIT numbers do not add to the total because clinics could report multiple FIT types

^**^USPSTF evidence review identified this FIT with adequate data to support high sensitivity and specificity

Of the 84 unique responses, only 78 provided answers in the “FIT/FOBT use and costs” section and only 23 respondents initially knew the cost of their FIT. After the additional research team follow-up, as displayed in Table 1, 40 practices (48%) ultimately provided information on the cost of their FITs. Thirteen (32%) of these practices received the FITs for free while 27 (68%) paid for the FIT kits; median reported cost of a FIT was $3.04, with a range from $0.83 to $6.41 per test. The remaining respondents did not know the cost of their FIT.

Characteristics of the 84 practices appear overall and by practices with known/unknown FIT costs appear in Table 2. The majority of responding practices had less than 10 providers (73%) and over half were located in rural or frontier regions (61%). Fifty-seven percent of respondents obtained their FITs from the Lab that processes the tests; otherwise FITs were procured directly from a FIT manufacturer’s Vendor (30%) or a Health System (such as a hospital system or health maintenance organization) (12%). There were no significant differences in preference for FIT or preferred CRC screening modality between practices with known/unknown FIT costs.

**Table 2:** Participating Practice Characteristics Overall and by Cost of FIT (Known, Unknown)

	Overall Respondents		Known Cost		Unknown Cost		
	N	%	N	%	N	%	p*
**Total N**	**84**	**(100)**	**40**	**(100)**	**44**	**(100)**	
**Practice size**							
Solo/partnership (1, 2)	10	(12)	8	(20)	2	(5)	0.031^a^
Small to medium (3–10)	51	(61)	25	(63)	26	(59)	
Large (> 10)	23	(27)	7	(18)	16	(36)	
**Geographic location**							
Frontier	10	(12)	6	(15)	4	(9)	0.61^a^
Rural	41	(49)	20	(50)	21	(48)	
Urban	33	(39)	14	(35)	19	(43)	
**Source for FIT/FOBT kits**							
Laboratory	47	(57)	17	(43)	30	(71)	0.001^a^
Vendor	25	(30)	20	(50)	5	(12)	
Health System	10	(12)	3	(8)	7	(17)	
**Practice Characteristics**							
Number of patient visits per week, mean (min-max)	427	(32–5250)	314	(32–1200)	554	(105–5250)	0.07^b^
Percent of patient panel covered by Medicaid/CHIP/OHP, mean (min-max)	30	(2–85)	30	(2–75)	30	(5–85)	0.93^b^
Current CRC screening rate, mean (min-max)	59	(10–97)	56	(23–85)	62	(10–97)	0.14^b^
Number of QI projects related to CRC screening in past 12 months, mean (min-max)	1	(0–6)	1	(0–3)	1	(0–6)	0.86^b^
CRC improvement as priority in year ahead (1 no priority, 10 highest priority), mean (min-max)	8	(1–10)	8	(1–10)	8	(5–10)	0.74^b^
**Preferred CRC screening modality**							
FIT/FOBT	7	(8)	3	(8)	4	(9)	0.96^a^
Colonoscopy	10	(12)	5	(13)	5	(11)	
Both (colonoscopy & FIT/FOBT)	67	(80)	32	(80)	35	(80)	
**Preference for FIT/FOBT** (1 = hate, 10 = love), mean (min-max)	7	(1–10)	7	(1–10)	7	(3–10)	0.18^b^

^*^p value from (a) Pearson’s chi-squared test or (b) Wilcoxon rank-sum test. Lower *p* values provide some evidence that the distributions of the characteristic in question differ between the “known” and “unknown” cost groups

Because of the high proportion of practices that did not know their cost of FIT, we examine the practice characteristics associated with FIT costs using descriptive analysis on the sub-sample of practices that knew their costs. While we cannot assume that practices with unknown FIT costs have the same distribution of costs as the known practices, we examined the relationship between practice size, source of FIT, and cost per study objectives with results described below.

### Source of FIT and cost relationship

Practice size and source of FIT were both significantly associated with knowledge of FIT costs (*p* = 0.031 and 0.001 respectively), see Table 2. However, when practice size and source are considered in the same model, practice size was no longer statistically significant. This is likely because practice size and source were related; 13.6% of large practices obtain kits from vendors compared to 34% of small to medium sized practices (2.5 times as likely) and 50% of solo/partnerships (3.67 times as likely; *p* = .026 for nonparametric test for trend). Thus, cost differences were primarily driven by source of FITs (i.e., supplied by a lab, vendor, or health system).

**Fig. 1:**
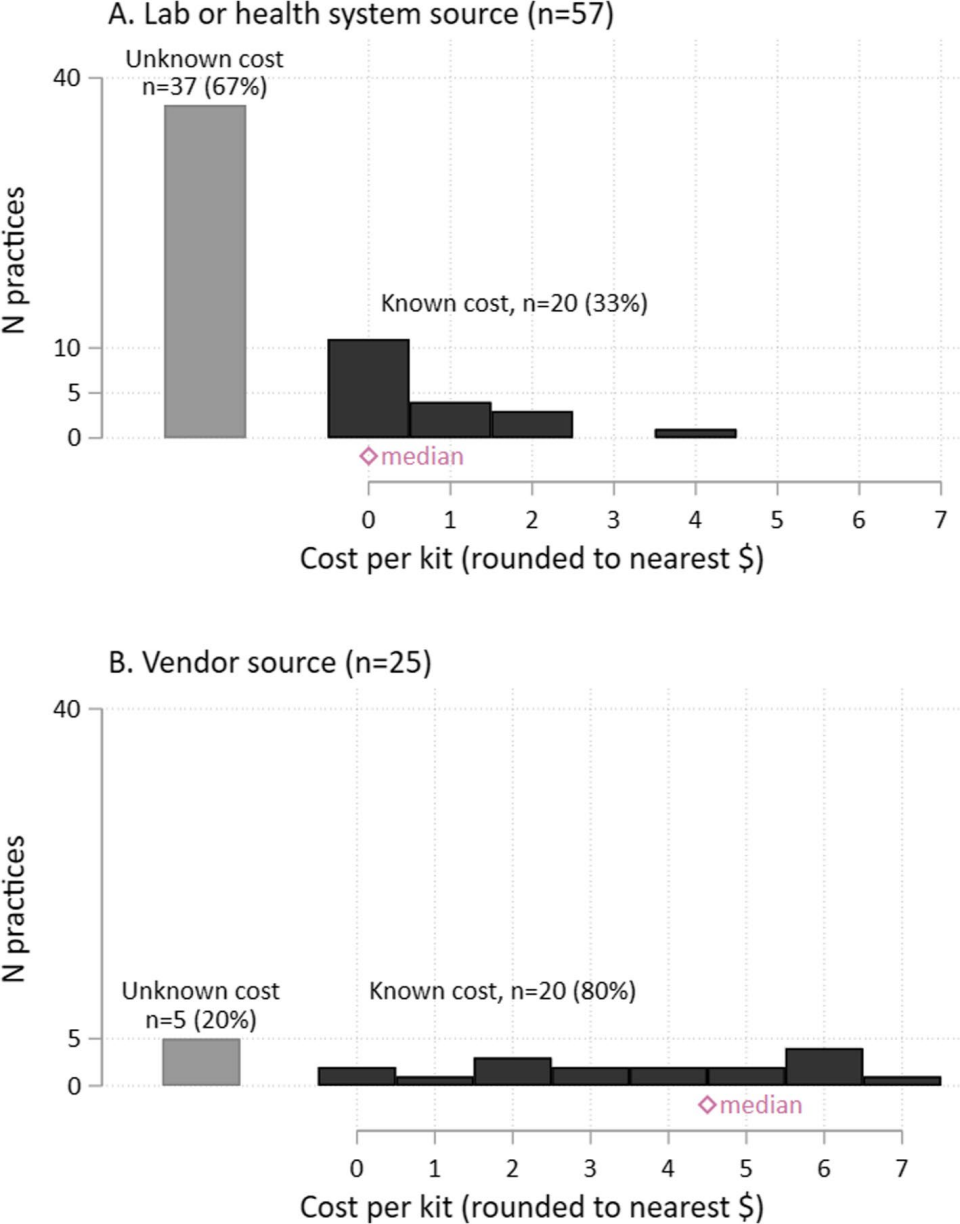
Cost Distribution by Source of FIT, in Practices with Known Cost*. * Practices who received FITs from vendors were more likely to know the cost (*p* = 0.0002) and, if known, had a higher cost (*p* = 0.0002). **a**. Lab or Health system source, *N* = 57. **b**. Vendor source, *N* = 25 practices. Grey bar = Unknown cost; Black bar = Known cost; Red diamond = Median reported cost

Practices who received FITs from vendors had a higher cost (*p* = 0.0002), and were more likely to know the cost (*p* = 0.0002). Costs were not significantly different by FIT type, even when we factored in source. In Fig. 1, we show the relationship between knowing the FIT cost and source of procurement (i.e., lab/health system vs. vendor), and in median FIT costs if known. We combined the lab and health system categories as sources, because health system was small and observed cost patterns were very similar to lab.

## Discussion

Our study was designed to explore how contextual factors (e.g., practice size, ownership, geographic location) were associated with use of two innovations to improve CRC screening (i.e., FIT and mailed FIT), with a primary goal of identifying factors influencing FIT costs. While 87% of the respondents knew the type of FIT in use at their clinical practice, only 40% could report on the costs; 32% of those reporting costs received their FITs for free. Of the practices reporting the cost of their FITs, we found no cost patterns by type of FIT or rurality. However, we identified a statistically significant relationship between cost and the *source* of the FIT such that labs and health systems are providing FITs for free or at low costs, while practices that procured FITs from a manufacturer’s vendor paid more.

Wide variation in FIT type and costs are not unique to our study, and have been found in prior demonstration projects and pragmatic trials [15, 44]. Such variation has the potential to impact a setting’s willingness and ability to implement effective evidence-based interventions such as mailed FIT. In alignment with the i-PARIHS framework [25], these variations in FIT type and costs suggested opportunities for practice facilitators to tailor support for clinics who are implementing mailed FIT programs. Thus facilitators may need to work with clinical team members to assess why a FIT is used, and to determine if more cost and clinically effective options are available.

For example, we found that only 43% of the practices surveyed were using FIT tests with strong evidence of clinical effectiveness for high sensitivity and specificity in the published literature (i.e., OC Auto®, OC Light®, Insure®) [45]. Of the 160 tests approved by FDA, the American Cancer Society recommends only about 10 of them [13] and the USPSTF evidence review identified only OC Auto® and OC Light® as having adequate data demonstrating high sensitivity and specificity [4]. This gap between recommended FITs and those used in routine practice suggest opportunities for further intervention and improvement during mailed FIT implementation.

Addressing the source, and thus cost of FIT, may also support mailed FIT implementation and program sustainability. Other studies have also found variation in the costs of FIT. One CRC screening demonstration program found more than a two fold variation in FIT costs between the two screening sites: from $3 per kit (Nebraska site, *n* = 1264 persons) to $7 per kit (Greater Seattle *n* = 867 persons) [44]. Variation in costs may be because larger systems with many clinical practices (such as a hospital system or a health maintenance organization) can find economies of scale for implementing screening outreach programs [31, 46]. Independent laboratories that process FIT tests can receive reimbursement for the FIT process, and therefore might be more willing to supply a FIT free of charge. However, given 60% of our respondents were unaware of FIT costs, it is possible that FIT purchasing decisions are not made by the primary care practice manager or quality improvement lead, or that the cost of the test is not typically a criteria for FIT selection. Thus facilitators may need to work with multiple levels within a setting to understand the factors impacting FIT source and selection in order to support changes to enable program implementation.

These findings should be considered within the larger context of addressing CRC screening disparities in small community practices. Over half of our survey respondents were located in rural or frontier regions of Oregon, and the majority had fewer than 10 providers. Rural areas are home to about 60 million people in the US, and Medicaid covers nearly 1 in 4 rural residents under age 65 (24%) [47], but rural and frontier residents have lower rates of cancer screening [48]. Many factors in this clinical context could impact the implementation of evidence-based practices. In this case, variation in FIT quality and the ability to obtain FITs in a cost-effective manner could lower the effectiveness or cost-effectiveness of a FIT screening outreach program. Cost fluctuations might have a larger impact on practices serving a smaller patient population. The Center for Disease Control and Prevention [49] found that, in cost data from 124 screening programs, those that screened a larger volume of people achieved a lower cost per person screened than those screening a smaller population and attributed the finding to economies of scale.

In the US health care system, mailed FIT programs are somewhat complicated to implement because costs for an individual clinical practice can vary greatly depending on who is purchasing FITs, where lab processing occurs, and who receives insurance reimbursement for completed FITs. For example, in the seven clinics in our sample that used multiple FIT tests in their practices, insurance coverage and health system factors led to their FIT selection. There are also external incentives for screening that could offset costs, such as state or federal incentive metrics. In addition, colonoscopy resource constraints and ensuring that colonoscopy capacity is sufficient to meet the demand for services is a consideration for any FIT outreach program [18, 46, 50]. One feature of interventions to implement FIT outreach programs should likely include working with practices to evaluate if changes are needed in their current FIT, and to advocate for tests that have clinical and patient preferred characteristics [14].

While the labor costs relating to FIT programs can be substantial, the variability in our findings shows that the cost of the FITs themselves should not be ignored. For some FIT outreach programs, FITs can be distributed only to patients who are more likely to complete them, such as handing them out in clinic. Patients generally have a higher chance of completing a FIT given to them by their provider in-person [51, 52] However, a mailed FIT program has the potential to reach more patients overall, but possibly with a lower *rate* of completion. Mailed FIT programs are known to increase CRC screening rates, in a range of anywhere from 15 to 28% [17, 19] and they are especially effective at reaching patient groups at higher risk for being unscreened [53, 54]. However, because of the structure of a mailed FIT program [29–31], the cost of individual FITs may make a large difference because FITs are mailed to everyone in a population. Clinical practices may have concerns regarding costs associated with distributing FITs that are not completed. Primary care practices might be more reluctant to purchase FIT kits for their entire population overdue for CRC screening. A small difference in the cost of FIT is magnified in this approach, such that FITs procured at the minimum cost reported here of $0.83 versus the maximum of $6.41 could lead to an almost 8-fold difference ($83 vs $641) if mailed FITs were distributed to 100 patients in a clinic.

By knowing the cost of the FIT tests themselves, primary care practices can better evalutate the return on investment of FIT outreach programs, and also whether investments in activities that increase rates of return would help offset the costs of unreturned FIT tests. A population-based FIT testing approach is even more effective when paired with interventions to decrease barriers to CRC screening, such as patient reminders, patient or provider incentives, education, or FIT mailing programs [16, 33]. Our results might make it easier for primary care practices to assess the cost-benefit of implementing these programs to increase rates of FIT returns.

### Limitations

Our study does have certain limitations. First, this was a cross-sectional survey of primary care practices within one state and there may be different cost patterns in the non-responding practices. However, our response rate of 33% is similar to prior surveys [55–58]. Second, our findings must be interpreted cautiously because a large number of respondents could not tell us the cost of their FITs, therefore we are not able to do regression analysis by clinical characteristics of the full sample. We cannot determine if the practices with unknown FIT costs have same distribution as known costs. For example, it is possible that the practices who did not know the cost of their FITs were mostly practices who had the FITs provided for free or at a low cost. Third, many of the FITs in use lack evidence of clinical effectiveness; yet they were clearly the preferred test for these clinical sites. Qualitative work, for example, could explore the relationships between practices, vendor, lab, and FIT selection to determine the driving factors as well as to explore why practices may still utilize poorer quality FIT/FOBTs. While surveys with validated measures are a good starting point to examine contextual factors influencing FIT selection and costs, this area of research would benefit from more qualitative research to identify other elements of organizational (inner) and outer context that might affect FIT costs and implementation of FIT programs. Finally, purchase of a certain type of FIT does not equate to the full costs of using FIT to screen patients, since it excludes lab processing costs and implemention of any labor or staffing for outreach programs. Our survey data does not let us explore when the purchase of the FITs are offset by lab processing reimbursements of those tests. In other words, there might be broader system-level cost considerations that a health care practice takes into account. Despite these limitations, primary care clinics could use these data to examine return on investment of various CRC screening outreach approaches (cost per test/cost per completed test) and to negotiate for FITs at a better price.

## Conclusions

A high percentage of practices in our survey sample were unable to report on the cost of their FIT tests. In the sub-group that knew the FIT test cost, we found a significant relationship between source of FIT and costs, rather than type of FIT. Primary care practices that purchase FITs from a vendor might need to spend more than practices that have FIT tests supplied by either a health system of which they are a part or the labs that process those FITs. Future research could examine how practices choose their FIT, whether practices are constrained to certain FITs by organizational purchasing restrictions, and how FIT cost specifically impacts visit-based test distribution as well as willingness to implement mailed outreach programs. Differences in the ability to obtain FIT tests in a cost-effective manner could have far reaching consequences for addressing CRC screening disparities in primary care practices.

## Supplementary Information

**Additional file 1.**

## Data Availability

The datasets used and/or analyzed for the current study are available from the corresponding author on reasonable request.

## Abbreviations

CRC: Colorectal cancer
FIT: Fecal immunochemical testing
EHR: Electronic health record
FOBT: Fecal occult blood test
ORPRN: Oregon Rural Practice-Based Network
QI: Quality Improvement
