# Supplementary material for: Source matters: a survey of cost variation for fecal immunochemical tests in primary care
Source: BMC Health Serv Res. 2022 Feb 15;22:204. doi: 10.1186/s12913-022-07576-4 (PMC8845335; doi:10.1186/s12913-022-07576-4)
Supplement: Supplementary file 1 — Additional file 1. [file 12913_2022_7576_MOESM1_ESM.pdf]

ORPRN
Oregon Rural Practice-Based Research Network

Appendix 1. Readiness Survey
Understanding Practice Readiness to Increase Colorectal Cancer Screening via Direct Mail Programs

Thank you for completing this ORPRN survey to better understand the reasons a practice may choose to change the way care is delivered.

- We focus on using direct mail programs to improve screening for colorectal cancer as a test case.

- We are interested in the views from practices that **have**, and **have not**, used direct mail programs.

- **This survey takes 8-15 minutes to complete and is broken into 5 sections.**

- This survey should be completed by a **leader in the practice** who has good insights into the clinical operations and changes underway, such as a medical director, office manager, or quality improvement specialist.

You may complete the survey electronically – or return hard copies by mail, fax, or email to:

Oregon Rural Practice-based Research Network
Oregon Health & Science University
3181 S.W. Sam Jackson Park Road, Mail Code: L222
Portland, OR 97239-3098

Fax | Email

If you have questions about the survey, or direct mail programs, please contact us at:

- Name, Project Manager | email
- Name, Principal Investigator | email
- Name, Research Assistant | email

The following information is provided for your reference:

- Annual testing using **Fecal Immunochemical Tests (FIT)** or high sensitivity **Fecal Occult Blood Tests (FOBT)** can help prevent and reduce **colorectal cancer (CRC)** in average risk patients aged 50-75.

- **Direct mail programs** are an evidence-based intervention where a FIT/FOBT is sent in the mail to the home of patients who are due for CRC screening. Direct mail programs can increase CRC screening by 6-40% depending on the associated materials, reminders, and population served.

- Some practices are partnering with labs, payers, or larger health systems to support their use of direct mail programs.

A fully operational direct mail program requires practices to:

- Choose to implement the program
- Identify a practice champion and improvement team
- Select and identify a FIT/FOBT for use in CRC screening
- Develop a process for identifying patients that are due for CRC screening
- Coordinate distribution of FIT/FOBT kits and associated materials to eligible patients
- Implement reminders to encourage test completion
- Reconcile laboratory test results
- Support patient follow-up to colonoscopy on abnormal screens
- Monitor and review CRC screening data to inform continued improvement

SECTION 1: BASICS

1) Practice Name: ____

2) Practice Address: ____

3) Approximately how many research projects or quality improvement initiatives has your practice worked on in the last 12 months? __________

4) Approximately how many of the research projects or quality improvement initiatives reported in #3 above, were related to CRC screening? __________

5) What is the preferred modality for CRC screening at your practice?
   a. Colonoscopy
   b. FIT/FOBT
   c. Both a and b
   d. Other, please specify: _____________________________

6) On a scale from 1 to 10 where one is hate it to 10 is love it, how do your clinicians generally feel about using FIT/FOBT for colorectal cancer screening? Circle one:

1	2	3	4	5	6	7	8	9	10
Hate FIT/FOBT……………………………………………………….Love FIT/FOBT									

Details?

7) On a scale from 1 to 10 where one is no priority at all and 10 is the highest priority, what is the priority that your practice's leadership places on improving CRC screening in the year ahead?

1	2	3	4	5	6	7	8	9	10
No priority……………………………………………………………highest priority									

8) What is your clinic's current up-to-date rate for CRC screening in age eligible members (50-75)?
   a. Rate: ______________________
   b. How determined, please specify (e.g., electronic health record, best guess):

   ____

SECTION 2: FIT/FOBT USE & COSTS

Work by our team suggests that FIT/FOBT costs vary widely across practices. We are trying to understand why costs vary, and how practices might use this information to negotiate/deliver high quality care. Please help us by answer the following questions:

9) What FIT/FOBT is your practice currently using? [check all that apply]
   a. OC Auto-FIT (Polymedco)
   b. OC-Light iFOBT Test (Polymedco)
   c. QuickVue iFOBT (Quidel)
   d. Hemosure One-Step iFOBT test (Hemosure, Inc)
   e. Insure FIT (Clinical Genomics)
   f. Hemmocult-ICT (Beckman Coulter)
   g. I don’t know
   h. Other, please specify: ____________________

10) Where do you get your FIT/FOBT kits from?
   a. Laboratory
   b. Vendor
   c. Health System
   d. Other, please specify: _______________

11) Do you know how much a FIT/FOBT kit costs your practice?
   a. No
   b. Yes, how much? ____

12) Do you know how much an uninsured patient at your practice is charged for completing a FIT/FOBT kit?
   a. No
   b. Yes, how much? ____

SECTION 3: DIRECT MAIL PROGRAM USE

13) When does your practice plan to begin using a direct mail program to increase CRC screening?
   a. We are <u>not planning</u> to use a direct mail program
   b. We <u>are planning</u> to use a direct mail program starting in (specify year): _______________
   c. We are <u>currently using</u> a direct mail program (specify year started): _________________

14) Did your practice work with any organizations/partners (e.g., American Cancer Society, your Coordinated Care Organization, a research team) to implement the direct mail program?
   a. Not applicable
   b. No
   c. Yes, specify who and how you work(ed) together:

15) On a scale from 1 (not at all) to 5 (fully), to what extent has your practice taken the following steps to use a direct mail program to increase CRC screening?

	Not at all	Considering	Started	Refining	Fully
a. Chosen to implement a direct mail program	1	2	3	4	5
b. Identified a practice champion and improvement team	1	2	3	4	5
c. Developed a process to identify patients due for CRC screening	1	2	3	4	5
d. Coordinated distribution of FIT/FOBT kits to patients	1	2	3	4	5
e. Implemented reminders to encourage test completion	1	2	3	4	5
f. Developed a process to reconcile lab results	1	2	3	4	5
g. Developed a process to support patient follow-up to colonoscopy on abnormal screens	1	2	3	4	5
h. Monitored and reviewed CRC screening data to inform continued improvement	1	2	3	4	5

For questions 16 and 17, please think <u>back to the time when your practice was considering using a direct mail program to increase CRC screening</u>. That may be where your practice is currently at, or it may be some time ago.

16) Our practice is ready to implement the changes needed for a direct mail program to increase CRC screening (circle one):

Disagree	Somewhat Disagree	Neutral	Somewhat Agree	Agree

17) Please circle the response that best reflects your <u>practice's</u> readiness to implement a direct mail program. *[Note: Some items may feel repetitive – we apologize. Our goal is to see if a single item can replace this existing multi-item instrument. Please help by completing all questions!]*

	Disagree	Somewhat Disagree	Neither Agree nor Disagree	Somewhat Agree	Agree
a. We are committed to implementing a direct mail program.	1	2	3	4	5
b. We can keep track of progress in implementing a direct mail program.	1	2	3	4	5
c. We will do whatever it takes to implement a direct mail program.	1	2	3	4	5

17) CONTINUED…Please circle the response that best reflects your practice's readiness to implement a direct mail program. *[Note: Some items may feel repetitive – we apologize. Our goal is to see if a single item can replace this existing multi-item instrument. Please help by completing all questions!]*

	Disagree	Somewhat Disagree	Neither Agree nor Disagree	Somewhat Agree	Agree
d. We can support providers as they adjust to a direct mail program.	1	2	3	4	5
e. We want to implement a direct mail program.	1	2	3	4	5
f. We can handle the challenges that might arise in implementing a direct mail program.	1	2	3	4	5
g. We are determined to implement a direct mail program.	1	2	3	4	5
h. We can coordinate tasks so that implementation of a direct mail program goes smoothly.	1	2	3	4	5
i. We are motivated to implement a direct mail program.	1	2	3	4	5
j. We can manage the politics of implementing a direct mail program.	1	2	3	4	5

SECTION 4: PRACTICE CHARACTERISTICS

18) Please provide the number of clinicians (MD, DO, NP, PA) and their combined full-time equivalent (FTE):
    a. Number clinicians: _________________________
    b. Combined clinician FTE: _____________________

19) Please estimate the total number of patient visits over a typical week at your practice: _________

20) Please estimate the percentage of your practice's current patient panel that is male: ______%

21) Please estimate the percentage of your practice's current patient panel that is Hispanic or Latino: _____%

22) Please estimate the percentage of your practices revenue from patient care that comes from:
    Commercial Insurance: _______%
    Medicaid/CHIP/OHP: ________%
    Medicare: ______%
    Other: ______%

23) If we have clarifying questions about your responses on this survey, what is your name and the best way we can reach you?

a. Name: ____

b. Phone: ____

c. Email: ____

d. What is your role in the practice: _____________________________________

SECTION 5: FINAL QUESTIONS (YOU'RE ALMOST DONE!)

24) To what extent has your practice used any of the following activities to increase CRC screening in the last 12 months?

a. Written reminders (letter, postcard, email) to tell patients they are due for screening	Never	Sometimes	Routinely	Unsure
b. Used telephone messages (automated, live) or text to tell patients they are due for screening	Never	Sometimes	Routinely	Unsure
c. Provided patient incentives (small rewards through cash or coupons) aimed to motivate patients to get screened or to encourage others (e.g., family members, close friends)	Never	Sometimes	Routinely	Unsure
d. Provided printed materials (e.g., letters, brochures, newsletters) or videos to inform and motivate patients to get screened – aka "small media"	Never	Sometimes	Routinely	Unsure
e. Used supportive staff (e.g., lay health advisors, clinical staff) to inform individuals about the need and benefits of screening, and helping overcome barriers.	Never	Sometimes	Routinely	Unsure
f. Presented clinicians/staff with information about their performance in providing screening	Never	Sometimes	Routinely	Unsure
g. Provided direct or indirect rewards (monetary or otherwise) to motivate providers to screen patients.	Never	Sometimes	Routinely	Unsure
h. Let clinicians/staff know a patient is due or overdue for CRC screening (reminder)	Never	Sometimes	Routinely	Unsure
i. Reduced patient's out-of-pocket costs for CRC screening (e.g., vouchers, changes to co-pay)	Never	Sometimes	Routinely	Unsure
j. Reduced administrative barriers (e.g., patient can request FIT or get it by mail)	Never	Sometimes	Routinely	Unsure

25) Please indicate the extent to which you agree or disagree that your practice has used the following strategies to improve colorectal cancer (CRC) screening:

a. Providing information and skills-training	Strongly Disagree	Somewhat Disagree	Neither Agree or Disagree	Somewhat Agree	Strongly Agree	N/A
b. Using opinion leaders, role modeling, or other vehicles to encourage support for changes	Strongly Disagree	Somewhat Disagree	Neither Agree or Disagree	Somewhat Agree	Strongly Agree	N/A
c. Changing or creating systems in the practice that make it easier to provide high quality care	Strongly Disagree	Somewhat Disagree	Neither Agree or Disagree	Somewhat Agree	Strongly Agree	N/A
d. Removing or reducing barriers to better quality of care	Strongly Disagree	Somewhat Disagree	Neither Agree or Disagree	Somewhat Agree	Strongly Agree	N/A
e. Using teams focused on accomplishing the change process for improved care	Strongly Disagree	Somewhat Disagree	Neither Agree or Disagree	Somewhat Agree	Strongly Agree	N/A
f. Delegating to non-clinician staff the responsibility to carry out aspects of care that are normally the responsibility of physicians	Strongly Disagree	Somewhat Disagree	Neither Agree or Disagree	Somewhat Agree	Strongly Agree	N/A
g. Providing to those who are charged with implementing improved care the power to authorize and make the desired changes	Strongly Disagree	Somewhat Disagree	Neither Agree or Disagree	Somewhat Agree	Strongly Agree	N/A
h. Periodic measurement of care quality for assessing compliance with any new approach to care	Strongly Disagree	Somewhat Disagree	Neither Agree or Disagree	Somewhat Agree	Strongly Agree	N/A

ORPRN Oregon Rural Practice-Based Research Network

26) [Last question!] Please indicate the extent to which you agree or disagree that your practice has used the following strategies to improve colorectal cancer (CRC) screening:

a. Reporting measurements of practice performance on CRC screening for comparison with their peers	Strongly Disagree	Somewhat Disagree	Neither Agree or Disagree	Somewhat Agree	Strongly Agree	N/A
b. Setting goals and benchmarking rates of performance quality on CRC screening at least yearly	Strongly Disagree	Somewhat Disagree	Neither Agree or Disagree	Somewhat Agree	Strongly Agree	N/A
c. Customizing implementation of CRC screening care changes to the practice	Strongly Disagree	Somewhat Disagree	Neither Agree or Disagree	Somewhat Agree	Strongly Agree	N/A
d. Using rapid cycling, piloting, pre-testing, or other vehicles for reducing the risk of negative results for introducing organizational-wide change	Strongly Disagree	Somewhat Disagree	Neither Agree or Disagree	Somewhat Agree	Strongly Agree	N/A
e. Deliberately designing care improvements so as to make clinician participation less work than before	Strongly Disagree	Somewhat Disagree	Neither Agree or Disagree	Somewhat Agree	Strongly Agree	N/A
f. Deliberately designing care improvements to make the care process more beneficial to the patient	Strongly Disagree	Somewhat Disagree	Neither Agree or Disagree	Somewhat Agree	Strongly Agree	N/A

Thank you for your time and participation! This information will be used to refine interventions to improve CRC screening and treatment.

For more information on FIT/FOBT options or help selecting a FIT, please see the ACS Issue Brief, Finding the Right FIT or contact us directly.
